# Being a morning man has causal effects on the cerebral cortex: a Mendelian randomization study

**DOI:** 10.3389/fnins.2023.1222551

**Published:** 2023-07-20

**Authors:** Fan Yang, Ru Liu, Sheng He, Sijie Ruan, Binghua He, Junda Li, Linghui Pan

**Affiliations:** ^1^Department of Anesthesiology, Guangxi Medical University Cancer Hospital, Nanning, Guangxi Province, China; ^2^Department of Anesthesiology, Central Hospital of Shaoyang, Shaoyang, Hunan Province, China; ^3^Guangxi Key Laboratory for Basic Science and Prevention of Perioperative Organ Disfunction, Nanning, Guangxi Province, China; ^4^Guangxi Clinical Research Center for Anesthesiology, Nanning, Guangxi Province, China; ^5^Guangxi Engineering Research Center for Tissue and Organ Injury and Repair Medicine, Nanning, Guangxi Province, China; ^6^Department of Anesthesiology, The First Affiliated Hospital of Southern China University, Hengyang, China

**Keywords:** Mendelian randomization, circadian rhythm, brain cortex, causal effect, morningness

## Abstract

**Introduction:**

Numerous studies have suggested a connection between circadian rhythm and neurological disorders with cognitive and consciousness impairments in humans, yet little evidence stands for a causal relationship between circadian rhythm and the brain cortex.

**Methods:**

The top 10,000 morningness-related single-nucleotide polymorphisms of the Genome-wide association study (GWAS) summary statistics were used to filter the instrumental variables. GWAS summary statistics from the ENIGMA Consortium were used to assess the causal relationship between morningness and variates like cortical thickness (TH) or surficial area (SA) on the brain cortex. The inverse-variance weighted (IVW) and weighted median (WM) were used as the major estimates whereas MR-Egger, MR Pleiotropy RESidual Sum and Outlier, leave-one-out analysis, and funnel-plot were used for heterogeneity and pleiotropy detecting.

**Results:**

Regionally, morningness decreased SA of the rostral middle frontal gyrus with genomic control (IVW: β = −24.916 mm, 95% CI: −47.342 mm to −2.490 mm, *p* = 0.029. WM: β = −33.208 mm, 95% CI: −61.933 mm to −4.483 mm, *p* = 0.023. MR Egger: β < 0) and without genomic control (IVW: β = −24.581 mm, 95% CI: −47.552 mm to −1.609 mm, *p* = 0.036. WM: β = −32.310 mm, 95% CI: −60.717 mm to −3.902 mm, *p* = 0.026. MR Egger: β < 0) on a nominal significance, with no heterogeneity or no outliers.

**Conclusions and implications:**

Circadian rhythm causally affects the rostral middle frontal gyrus; this sheds new light on the potential use of MRI in disease diagnosis, revealing the significance of circadian rhythm on the progression of disease, and might also suggest a fresh therapeutic approach for disorders related to the rostral middle frontal gyrus-related.

## 1. Introduction

Being either a morning or night person indicates the state of the circadian clock, a hierarchical network in mammals (Hu et al., 2016). The suprachiasmatic nucleus (SCN) in the hypothalamus acts as the main part of the central circadian clock, reactive with light and capable of resetting and containing the synchronous rhythm in peripheral tissues (Baxter and Ray, 2020). The innate circadian clock functions as a commander to coordinate physiological processes, like immunity, metabolism, and inflammation (Shivshankar et al., 2020) Disruption of the clock could be a risk for multiple outcomes, like obesity, diabetes, hyperlipidemia, cancer, and so on (Neves et al., 2022).

There is some solid evidence for a connection between circadian rhythm disorder and neurologic diseases such as Alzheimer’s and Parkinson’s disease (Gu et al., 2015; Weissova et al., 2016; Richmond and Davey Smith, 2022). It is also linked to mental illnesses such as bipolar disorder, depression, and anxiety, and to disorders such as autism (Walker et al., 2021; Martinez-Cayuelas et al., 2022; Takaesu et al., 2022; Ketchesin et al., 2023).

In addition, neurological diseases and psychosis frequently manifest with cognitive and consciousness impairments, varying in type and severity. Since the cerebral cortex is universally regarded as an advanced hub for cognition and consciousness, many neurological diseases and psychosis have been definitively linked to substantial structural alterations within the cerebral cortex. Examples include schizophrenia patients who had significant variations in 55 regions of cortical thickness, 23 regions of the volume, seven regions of the area, and 55 regions of local gyrification index than healthy individuals (Han et al., 2023). Children with autism spectrum disorder also experience a higher empathizing-systemizing difference than children without autism, which shares a significant negative association with the gyrification in the left lateral occipital cortex (Pan et al., 2023). Mild traumatic brain injury patients with posttraumatic headache experience a reduced headache impact and improved cognitive function in the acute to subacute phase, along with the restoration of cortical thickness of the left caudal anterior cingulate cortex and left insula and cortical surface area of the right superior frontal gyrus, and the restoration confirmed a key regulatory role by further mediation analysis (Xu et al., 2023). Parkinson’s Disease was discovered to have an initial presentation of thinner occipital, parietal, and temporal cortices, extending toward rostrally located cortical regions with increased disease severity, after which the bilateral putamen and amygdala shrink consistently, during which poorer cognition is connected with widespread cortical thinning and lower volumes of core limbic structures (Laansma et al., 2021). In Vuksanovic’s study, it was discovered that patients with Alzheimer’s Disease bear the most severe cognitive impairment, while individuals with behavioral variant Fronto-Temporal Dementia exhibit more impairment compared to healthy elders. Meanwhile, patients with Alzheimer’s disease and behavioral variant Fronto-Temporal Dementia maintain distinct patterns of cerebral cortex structural disturbance which deviate significantly from the changes observed in normal aging (Vuksanovic et al., 2019). It is also widely accepted that the entorhinal cortex act as the first brain area related to pathologic changes in Alzheimer’s disease (Li et al., 2021).

The exploration of the links among alterations in cerebral cortex structures, cognition, and consciousness has long been a focal point of scientific interest, which may advance human understanding of our cerebral cortex. One of the aims of this study is to explore the cortical changes induced by circadian rhythm disorder in humans, providing a structural localization for future investigations into the impact of circadian rhythms on cognitive function and serving as a filter in the analysis of large-scale imaging data such as MRI to figure out how much the circadian rhythm matters in these kinds of diseases. Additionally, a potential adjunctive therapeutic approach may be possible, targeting the specific cortical changes—improving/stabilizing circadian rhythms—although further validation is still required. To achieve the goal, it is not sufficient to merely identify the correlation between cortical structure and circadian rhythm since it is still unclear whether circadian rhythm disorders lead to changes in the brain or if the structural changes in the brain impact the rhythmic behavior in verse.

Mendelian randomization (MR) has recently received a lot more attention for its ability to infer credible causal relationships between exposures and outcomes (Richmond and Davey Smith, 2022). By fully utilizing the random assortment of genetic variants during the maturation of germ cells, MR analysis could gather the genetic variations relatively independently of environmental factors as instrumental variables (IVs), using it to divide the outcome cohort into subgroups, reaching the goal of randomization (Burgess and Thompson, 2015). Since the random assortment of genetic variants occurs before birth, a long time before the outcome onset, MR analysis has the ability to minimize biases of residual confounding and reverse causation (Bowden and Holmes, 2019).

Considering the uncertain causal association between being a morning person and the cerebral cortex, we performed an MR analysis to address this, aiming at exploring the related gyrus for further analysis and research.

## 2. Methods

### 2.1. Study design

In this study, a two-sample MR analysis was carried out to examine the causal effect between being a morning person and the structural changes of the cerebral cortex. The basic three MR assumptions urged to be met were (1) IVs should be in a robust correlation with exposure, (2) IVs should not respond to potential confounders, and (3) IVs could never affect the outcome except through exposure. To fulfill the assumptions, first, Europeans were determined to be the major subjects to minimize the potential confounders of ancestry. Then, cohorts in exposure and outcome were carefully selected to avoid the overlap of participants. Third, the PhenoScanner^1^ was chosen to be the reference library for ruling out related genetic variants.

### 2.2. Exposure GWAS-being a morning person

In this study, the top 10,000 morningness-related single-nucleotide polymorphisms (SNPs) of the Genome-wide association study (GWAS) summary statistics were used for a two-sample MR analysis (Hu et al., 2016). GWAS for morningness was performed on the 23andMe participant cohort (Eriksson et al., 2010). Morningness was defined by asking the participants twice if they are naturally night people or morning people in surveys that were either comprehensive with multiple questions on a subject matter or quick questions. Using the responses, participants were classified as night people, morning people, or missing. If one answer was missing, the other answer was used as the phenotype value. If one answer indicated being a morning person but the other indicated being a night person, the phenotype value would be treated as missing. As a result, 89,283 individuals were divided into morning person or night person, with the relatives dropped out, and European ancestry accounting for over 97%, referring to the European populations in threeHapMap 2 (Falush et al., 2007). Then, age, gender, and the top five PCs were used as covariates to account for population structure. Ultimately, Genome-wide Complex Trait Analysis (Yang et al., 2011) revealed that genetic variants could account for approximately 21% (95% CI: 13 to 29%) of the variability in the likelihood of being a morning person.

### 2.3. Outcome GWAS-the cerebral cortex

The brain cerebral cortex structural GWAS data was obtained from the ENIGMA Consortium (Grasby et al., 2020). In total, 51,665 individuals joined the project, nearly 94% of which were born with European ancestry, coming from 60 cohorts all over the world. Cortical measurements of surface area (SA) and thickness (TH) were obtained from brain magnetic resonance imaging (MRI) scans. The scans were segmented into 34 regions based on the Desikan-Killiany atlas (Desikan et al., 2006), resulting in a total of 70 phenotypes, including both regional and global SA and TH. Firstly, Grasby’s group filtered out 33,992 participants of European ancestry from cohorts for the next analysis to control the biases (cohort information was listed in Supplementary Table S1). Secondly, GWAS analyses were independently performed on the 70 imaging phenotypes, namely 34 measurements for TH, 34 measurements for SA, total TH, and total SA. In regional GWAS analysis, global measures of SA and TH were used as covariates. Finally, once quality control was finished, the data were meta-analyzed using METAL (Willer et al., 2010). As a result, 34% (SE = 3%) of the variation in total surface area and 26% (SE = 2%) in average thickness could be explained by common genetic variants in the GWAS summary statistics.

### 2.4. Selection of genetic instruments

**Figure 1:**
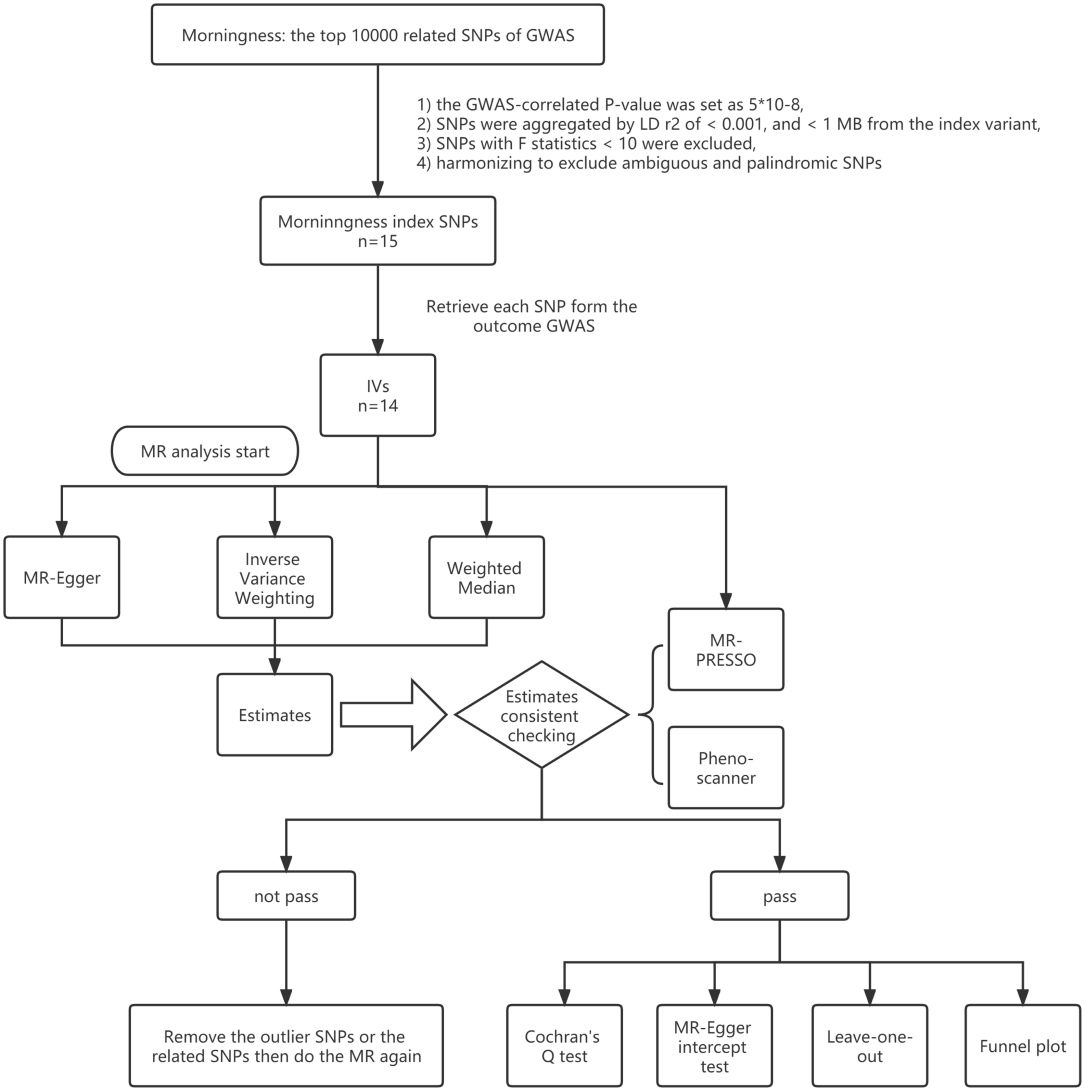
Study flame chart of the Mendelian randomization study to reveal the causal relationship between morningness and the brain cortical structure.

Genetic instruments were filtered with the following criteria: (1) the GWAS-correlated value of p was set as 5*10^−8^; (2) SNPs were aggregated by linkage disequilibrium (LD) r2 of <0.001, and < 1 MB from the index variant; (3) considering F statistic was able to quantify the strength of genetic instruments, SNPs with F statistics <10 were excluded; (4) harmonizing processes were conducted next to exclude ambiguous and palindromic SNPs; (5) after a first MR analysis, MR-pleiotropy residual sum and outlier (MR-PRESSO) process was used to check the SNPs with potential pleiotropy and to summarize a new value of *p* with outlier-corrected by removing the potential pleiotropy related SNPs, was after which, based on the new value of *p* and distortion test value of *p*, it was determined whether a second MR analysis should be necessary to verify robustness; and (6) PhenoScanner was used to see if the SNPs connected with the potential risk factors, including body mass index, obesity, smoking, drinking, neuropsychiatric disease, hypertension, and hyperlipemia, after which a second MR analysis would begin after removing SNPs associated with any of these potential confounders (Figure 1).

### 2.5. Mendelian randomization analyses

This study used random-effect inverse-variance weighted (IVW), MR-Egger, and weighted median as the major MR methods to address the variant heterogeneity and the pleiotropy effect. There are three assumptions that IVW needs to reach: (1) genetic variates need to be highly associated with exposure (Relevance hypothesis), (2) genetic variates should have nothing to do with confounding factors (Independence hypothesis), and (3) genetic variates could affect the outcome by exposure but nothing else (Exclusivity hypothesis). To sum up, it would be biased even if only one genetic variant is invalid (Burgess et al., 2013). Different from IVW, MR-Egger could be more robust to pleiotropy, and broadly used by allowing IVs to have a pleiotropic effect because there are only two assumptions for MR-Egger: (1) pleiotropic effect of IVs for outcome ought to be independent of the association between IVs and exposure, which is called the instrument strength independent of direct effect (InSIDE), relatively relaxing the constriction of exclusivity hypothesis (Bowden et al., 2015); and (2) there should be no measurement error (NOME) in correlation analysis between IVs and exposure. On the other hand, since the weighted median method uses the median IVs, it is robust to pleiotropy when >50% of the weight comes from valid IVs, which would result in a smaller type I error rate than IVW (Bowden et al., 2016). In the present study, IVW was chosen to be the main statistical method, because the cortical cortex structure-related SNPs were removed, outliers identified with MR-PRESSO were checked, and the InSIDE assumption of MR-Egger was difficult to confirm. Meanwhile, MR-Egger and weighted median were used to test the IVW estimates’ robustness as they could provide more robust estimates in a broader set of scenarios, despite being less efficient (wider CIs). Taking everything into account, it would be defined as robust in the present study when the statistics matched the following criteria: (1) the estimates from IVW and weighted median were both statistically significant, for the MR-Egger was so strict in bias control that it increased type II error rate inverse; and (2) BETA, which could be regarded as the slope of the regression of the SNP-outcome effects on the SNP-exposure effects, should be positive or negative at the same time in the three methods. Once the BETAs were inconsistent, a tightened instrument value of *p* threshold would be used, and then the MR analysis should be re-performed (Chen et al., 2020).

For significant estimates, the MR-Egger intercept test and leave-one-out analyses were used for further assessing horizontal pleiotropy. To identify heterogeneity, Cochran’s Q test was checked also.

### 2.6. Statistics

All analyses were performed using the packages TwoSampleMR (version 0.5.6) and MRPRESSO (version 1.0) in R (version 4.2.2) packages. Considering there were 136 MR estimates for region-level analyses and four MR estimates for global-level analyses, a Bonferroni-corrected value of *p* was set as 0.05/140 (3.57*10^−4^), while a value of *p* less than 0.05 and greater than 3.57*10^−4^ was considered nominally significant.

## 3. Results

There were 15 SNPs recognized for morningness (Supplementary Table S2), but rs3972456 was missing in the outcome-related GWAS summary statistics. The remaining 14 SNPs passed the filter procedures and were selected as IVs preliminarily, with F statistics for these genetic instruments all larger than the selected value 10, indicating a strong prediction.

Then, a comprehensive MR study was conducted to determine whether genetically predicted morningness connected with the 70 phenotypes with or without genomic control (GC). On a global level, no evidence existed for morningness causing changes in total SA or total TH, and the value of *p*s for the MR-Egger intercept were > 0.05. But when testing the causal relationship of morningness and total SA with/without GC, heterogeneity was observed with an IVW-related Cochran Q-derived *p*-value <0.05, and outliers were identified with MR-PRESSO global-test *p*-value <0.05, which could be explained as acceptable by the use of the random-effects IVW as the main result and unnecessary for a second MR analysis because of the MR-PRESSO outlier-corrected *p*-value and distortion-test *p*-value >0.05. On a regional level, it was the only estimate of nominal significance that morningness decreased SA of the rostral middle frontal gyrus (RMFG) with GC (IVW: β = −25.647 mm, 95% CI: −47.794 mm to −3.499 mm, *p* = 0.023. Weighted median: β = −34.769 mm, 95% CI: −47.794 mm to −6.550 mm, *p* = 0.016. MR Egger: β < 0) and without GC (IVW: β = −25.971 mm, 95% CI: −62.986 mm to −4.349 mm, *p* = 0.019. Weighted median: β = −33.677 mm, 95% CI: −62.509 mm to −4.845 mm, *p* = 0.022. MR Egger: β < 0), with the *p*-values for MR-Egger intercept >0.05, and no heterogeneity or outliers (Supplementary Tables S3–S6). For the remaining estimates on the regional level, it was not necessary to run a second MR analysis, even though heterogeneity was observed in some cases but no outliers were found or the MR-PRESSO outlier-corrected *p*-values were > 0.05.

**Figure 2:**
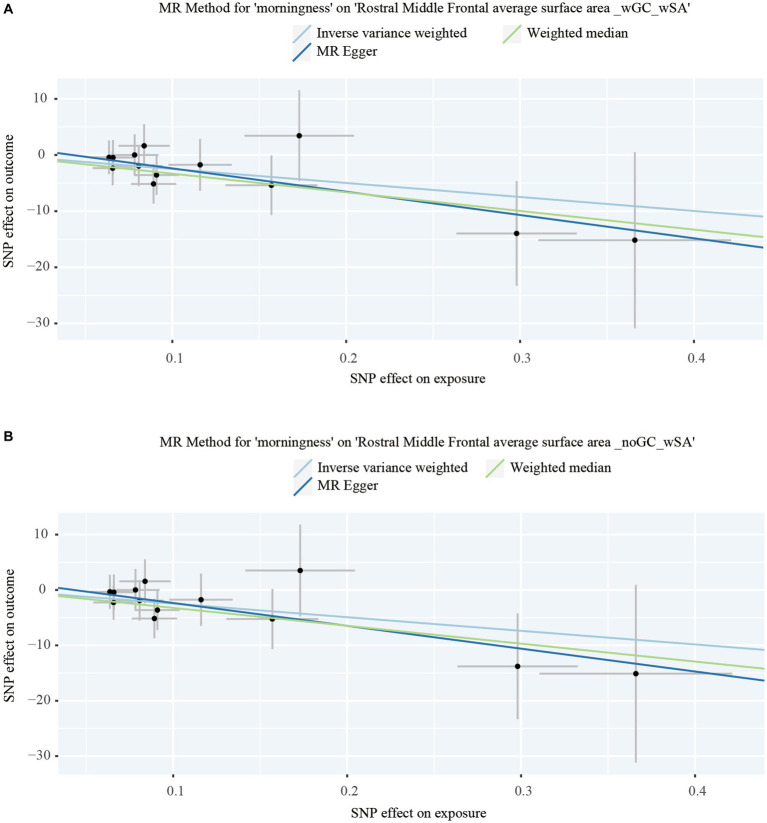
Dot plots of the significant Mendelian randomization statistics. **(A)** Statistics of morningness on the rostral middle frontal average surface area with genomic control applied and the total surface area included as a covariate; **(B)** statistics of morningness on the rostral middle frontal average surface area with the total surface area included as a covariate but genomic control not applied.

To figure out whether the nominal significant estimate could be violated by risk factors, Phenoscanner was used to check the IVs, finding that SNP rs13394871 was associated with body mass index (BMI), while others were associated with chronotype (Details of the instrumental variables are displayed in Supplementary Tables S7, S8). After removing rs13394871, estimates were consistent with the previous result. No causal relationship was verified on a global level, while on a regional level, only the nominally significant estimate remained, that morningness decreased SA of the RMFG with GC (IVW: β = −24.916 mm, 95% CI: −47.342 mm to −2.490 mm, *p* = 0.029. Weighted median: β = −33.208 mm, 95% CI: −61.933 mm to −4.483 mm, *p* = 0.023. MR Egger: β < 0) and without GC (IVW: β = −24.581 mm, 95% CI: −47.552 mm to −1.609 mm, *p* = 0.036. Weighted median: β = −32.310 mm, 95% CI: −60.717 mm to −3.902 mm, *p* = 0.026. MR Egger: β < 0), with the *p*-values for MR-Egger intercept >0.05, and no heterogeneity or outliers (Table 1, Figure 2 and Supplementary Tables S9–S12), indicating that the causal relationship between morningness and SA of the RMFG decreasing was not violated by potential risk factors.

**Table 1:** Significant Mendelian randomization estimates and the stability test results on the causal relationship of morningness and genetically predicted cortical structure.

Estimates		Rostral middle frontal average surface area _noGC_wSA	Rostral middle frontal average surface area _wGC_wSA
Inverse variance weighted (IVW)	N	13	13
	*p*-value	0.02943364	0.03596496
	SE	11.44181	11.71996
	BETA	−24.91603	−24.58055
	95% CI (BETA)	−47.34198 ~ −2.490075	−47.55168 ~ −1.609424
	OR	1.51*10^−11^	2.11*10^−11^
	95% CI (OR)	2.75*10^−21^ ~ 0.08290378	2.23*10^−21^ ~ 0.20000277
MR Egger	N	13	13
	*p*-value	0.1593967	0.1711283
	SE	27.49301	28.15703
	BETA	−41.49536	−41.22712
	95% CI (BETA)	−95.38165 ~ 12.39093	−96.41489 ~ 13.96066
	OR	9.52*10^−19^	1.25*10^−18^
	95% CI (OR)	3.77*10^−42^ ~ 240610.1	1.34*10^−42^ ~ 1156210.5
Weighted median	N	13	13
	*p*-value	0.02345826	0.02579779
	SE	14.65553	14.49354
	BETA	−33.20779	−32.30962
	95% CI (BETA)	−61.93263 ~ −4.482948	−60.71697 ~ −3.902274
	OR	3.78*10^−15^	9.29*10^−15^
	95% CI (OR)	1.27*10^−27^ ~ 0.01130005	4.28*10^−27^ ~ 0.02019594
Q-*p* value	IVW	0.9856199	0.9816331
	MR Egger	0.9833395	0.9789087
Pleiotropy	Egger intercept	1.772061	1.764273
	*p*-value	0.5289046	0.5208585
MR_PRESSO	Outlier corrected p	0.4098818	0.4041901
	Global Test p	0.0075	0.0033
	DistortionTest p	0.6848	0.6911

The significant estimate is defined as *p* < 3.57*10^−4^ in IVW and weighted median, and the nominal significant estimate is defined as *p* < 0.05 in IVW and weighted median. *N*, number of SNPs; Q-*p* value, Cochran’s Q-derived *p* value; noGC, without genomic control applied; wSA, the total surface area included as a covariate.

**Figure 3:**
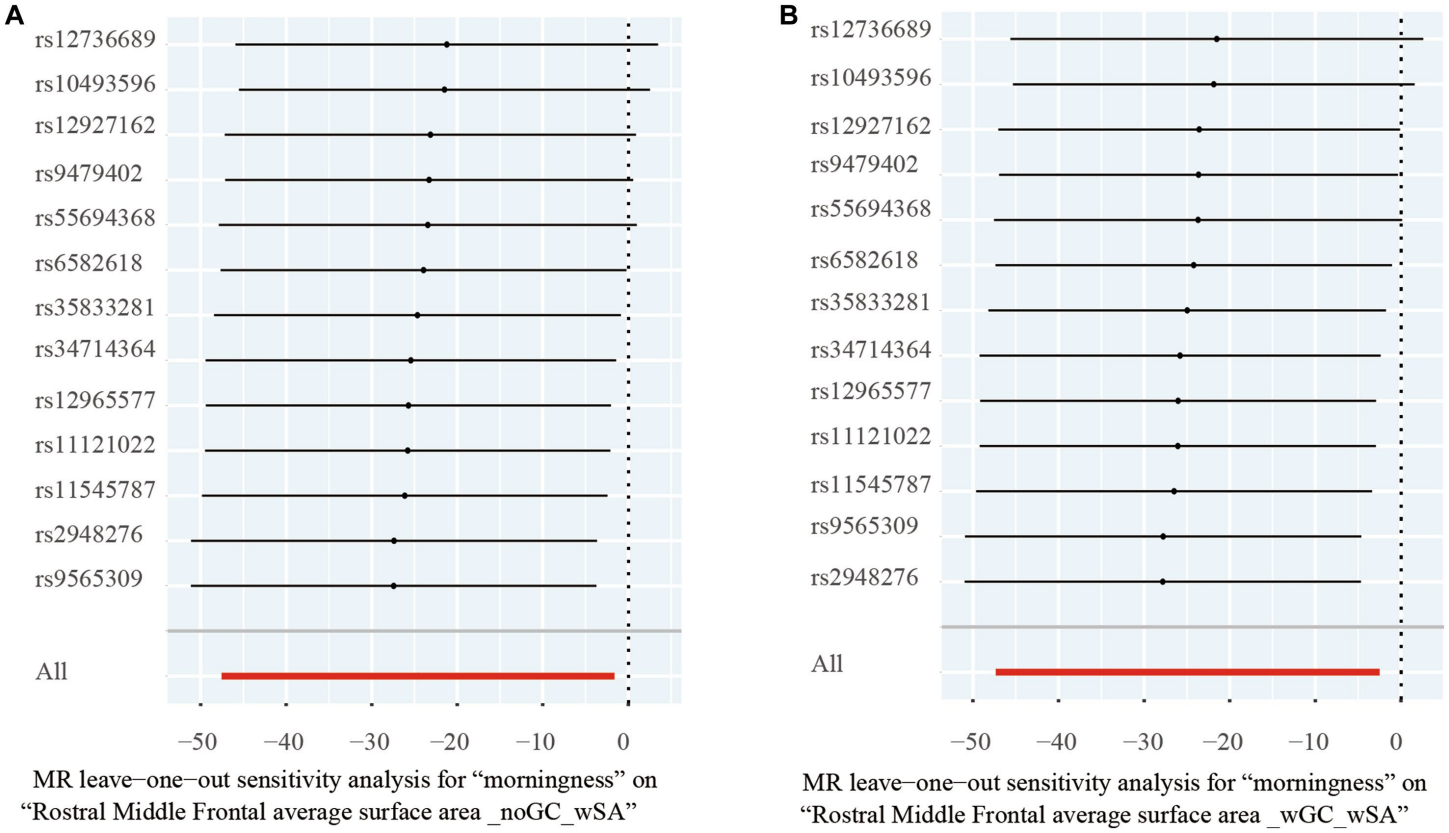
Leave-one-out analysis of the significant Mendelian randomization statistics. **(A)** Statistics of morningness on the rostral middle frontal average surface area with genomic control applied and total surface area included as a covariate; **(B)** statistics of morningness on the rostral middle frontal average surface area with the total surface area included as a covariate but genomic control not applied.

For significant estimates, leave-one-out analyses were used for further assessing horizontal pleiotropy, suggesting the estimates were not violated and biased by every single SNP (Figure 3).

## 4. Discussion

To the best of our knowledge, this is the first time a large-scale MR analysis has been carried out on the causal relationship between the state of circadian rhythm and changes in brain structure. Our study supports the causal relationship and provides novel information on the regional alterations ascribed to circadian rhythm.

Chronobiology began with Kleitman discovering its existence and is defined by Horne and Ostberg’s questionnaire (Kleitman, 1939; Horne and Ostberg, 1976). In Hu’s study, which supplied the exposure GWAS data source in the present study, to be a morning person is used as a substitute for rhythm following the steps of light, while a night one refers to a state in the opposite (Hu et al., 2016). Meanwhile, the present study points out that morningness decreases SA of the RMFG on a nominally significant level by systematically assessing the causality of morningness and cerebral cortex. That is to say, circadian rhythm disorder may cause an increase in rostral middle frontal gyrus surface area, but we cannot summarize a clear conclusion on whether the SA and TH of other gyri could be increased or decreased at the same time because the *p*-value of the causal relationship between morningness and SA of the RMFG is less than 0.05 but cannot pass the Bonferroni correction.

As the radial unit hypothesis posits, the increase of cortical SA is driven by the proliferation of neural progenitor cells, whereas TH is determined by the neurogenic divisions, which is a widely accepted hypothesis in neurology (Rakic, 1988, 1995, 2009), Grasby’s study goes further to suggest that SA is affected by genetic variants located in the regulatory element of progenitor cells in fetal brain tissue rather than adult brain tissue, whereas TH is associated with the regulatory elements which are close to genes implicating cell differentiation, migration, adhesion, and myelination in adult brain tissue (Grasby et al., 2020). In other words, SA is more innate, while TH is determined later and environmentally. Consequently, it becomes mainstream to use SA and TH as the basic neuroimaging instruments to discover underlying neurobiological mechanisms of both congenital and acquired diseases. It seems to be that SA and TH are highly heritable, of which the variants represent a pathological process from different periods in neurodevelopment (Strike et al., 2019).

However, it turns out to be the environmental factors that are more important. Whether through interventional studies or observational studies, whether it be normal physiological alterations, acute trauma, or psychogenic diseases with complex etiology and mechanisms, it has been verified that SA and TH both exhibit changes in response to environmental variations. For example, Voineskos has conducted a randomized placebo-controlled clinical trial clarifying the effects of antipsychotic medication on brain structure, finding that those who relapse receiving a placebo experienced decreases in cortical thickness compared with those who sustain remission (Voineskos et al., 2020). And Besiroglu has examined cortical TH and SA in 30 patients with obsessive–compulsive disorder (OCD), 21 unaffected siblings (SIB), and 30 controls, finding that both OCD and SIB groups show significantly lower cortical thickness in the right anterior insula compared to healthy controls and there are no significant differences in cortical thickness and surface area between the OCD and SIB groups (Besiroglu et al., 2022). When it comes to the normal physiological alterations, scientists point out that during pregnancy, an obvious change in cortical TH is discovered in mothers, suggesting brain structural adaptations for maternal love behavior (Hoekzema et al., 2017; Kim et al., 2018; Carmona et al., 2019; Zhang et al., 2019). Meanwhile, a study of 30 patients with acute mild traumatic brain injury (mTBI) and 27 matched healthy controls discovered a significant increase of cortical SA and volume in the right lateral occipital gyrus of acute-stage mTBI patients, but no significant changes in TH, which might explain a compensatory mechanism for cognitive dysfunction in acute-stage mTBI patients (Li et al., 2022).

In the Brodmann partition, RMFG refers to a part of the region on the outer superior surface of the frontal lobe that includes the superior and middle frontal gyri (Amunts and Zilles, 2015). The superior frontal gyrus is associated with advanced cognitive functions such as decision-making, reasoning, and planning, while the middle frontal gyrus is located posteriorly to the superior frontal gyrus and is also known as the precentral gyrus, involved in the planning, execution, and maintenance of working memory (Badre and D’esposito, 2009; Krudop and Pijnenburg, 2015). Therefore, the RMFG plays an important role in cognitive control and planning, and is shown to be associated with many neurological and psychiatric disorders such as attention deficit hyperactivity disorder, Alzheimer’s disease, and schizophrenia. For example, in the research on depression in Parkinson’s patients, it was found that thinning of the RMFG cortical area is associated with worsening depression. Many scientists have shown that the thickness of cortical layers in the RMFG region correlate with familial Alzheimer’s disease, depression severity in Parkinson’s patients, and first-episode schizophrenia (Quan et al., 2013, 2020; Yin et al., 2022).

The RMFG is also regarded as an emotion regulation center (Koenigs and Grafman, 2009). Emotion sensitivity is verified in Leon’s study, where he has followed the examination of 24 mothers who neglect their children (the neglect group, NG) and 21 mothers with non-neglectful caregiving (21 in the control group, CG); he discovers that there is a decrease in TH of the right rostral middle frontal gyrus and an increased SA in the right lingual and lateral occipital cortices for the NG (Leon et al., 2021). There are other existing forms of emotion regulation disorders as well. In a bipolar disorder-related study, the severity of the disease corresponds with the thinner TH of the RMFG, but an increase of the TH on the RMFG is discovered after a period of treatment (Hibar et al., 2018). And Hibar’s research also answers the question on whether RMFG could be influenced by the postnatal environment. While most of the studies above are cross-section experiments, a prospective study reached the same answer that the SA of RMFG could be influenced by the postnatal environment by demonstrating that, with no difference in the childhood brain structure, socioeconomic status in childhood is associated with adulthood brain structure changes in cortical TH of the precentral gyrus, postcentral gyrus, and caudal middle frontal, and in cortical SA of the RMFG, caudal middle frontal gyrus, and superior frontal gyrus (Dufford et al., 2021). All the SES-related variations have been further related to differences in cognitive, affective, and socio-emotional outcomes (Johnson et al., 2016; Dufford et al., 2019).

Our research mainly focused on the impact of circadian rhythms on brain function. Based on the results of the present study, it was found that circadian rhythms are indeed causally related to the SA of RMFG. A pilot study, fitting with the present study to some degree, indicates that 40 min of aerobic exercise and 20 min of anaerobic exercise a day, maintaining for 6 months, increases cortical thickness in the left pericalcarine area, the left superior parietal area, right RMFG, and right lateral occipital gyrus (Bashir et al., 2021), as physical exercise is beneficial to mental health and sleep (Hartescu et al., 2015; Panagiotou et al., 2021). Due to the impact of NO2 pollution on illumination, a cross-sectional study shows that exposure to air pollution of NO2 is associated with brain structure variates, including increases in the RMFG volume, supramarginal gyrus volume, the transverse temporal volume of the left brain, and the pars opercularis volume of the right brain (Lo et al., 2022), and air pollution exposure of NO2 is also associated with an increase in arousal (Lo et al., 2021; Tung et al., 2021).

Most of the variates in cortical structure observed in neurological disorders and diseases have been reported for TH, perhaps suggesting that TH would be more sensitive to environmental factors, such as treatment, illness, and so on (Grasby et al., 2020). The present study defines a significant estimate relating to SA, but not TH, which could be explained by the phenotypic variance of the RMFG being affected by common variants more for SA (21%) than TH (6%) (Grasby et al., 2020). In addition, changes in SA should not be viewed as a miracle, in other words, they may necessitate a longer duration like in Dufford’s study (Dufford et al., 2021), reflecting a chronic process, but they do make sense. Eero Vuoksimaa’s study suggests the same, wherein multivariate genetic analysis of 515 pairs of middle-aged twins reveals that the phenotypic and genetic association between neocortex volume and general cognitive ability is mainly driven by surface area rather than thickness (Vuoksimaa et al., 2015).

Notably, the present study confirms an estimate varied from the logical expectation. Being a morning person should be beneficial to our health as this suits the diurnal rhythm well, but in the present study, morningness was found to decrease the SA of the RMFG. Stomby’s cross-sectional prospective study might have served an explanation with sleepless, by indicating that higher cortisol in elderly people with less sleep correlates with the decrease of SA on the RMFG (Stomby et al., 2016). However, for night people, compensatory hypertrophy could explain the increased SA of the RMFG, the same as the consequence of Li’s study pointing out that patients with brain injury are discovered to have a significant increase of cortical SA and volume in the right lateral occipital gyrus (Li et al., 2022). All the evidence leads us to believe that the effects of circadian rhythm on the brain cortex may extend beyond what we currently understand, and urges further studies to elucidate the mechanism.

However, the core assumptions for MR analysis persist: (1) IVs need to be highly associated with exposure, (2) IVs should have nothing to do with confounding factors, and (3) IVs could affect the outcome by exposure but nothing else, MR analysis is faced challenges at its roots. Since the total phenotypic variation of the segregating population (VP) equals the sum of phenotypic variation coming from genetic variation (VG), variation related to environment factor (VE), and variation associated with the genetic and environmental factor interactions (VGE) (Andrew et al., 2010), there would be a statistical loss in the study using genetic variates as IVs for representing the phenotype of exposure.

In the present study, it was found that IVs could only account for approximately 21% of the morningness phenotype (Hu et al., 2016), but they still exhibited a nominally significant causal relationship with the decrease of SA in the RMFG. In other words, due to the high specificity and low sensitivity of the IVs, the conclusions derived from the present MR analysis have a low Type I error rate and a high Type II error rate. In layman’s terms, even though only around 21% of the participants were accurately identified (VG’s contribution to VP), the impact of circadian rhythm on RMFG still reached the threshold of nominal significance, and underdiagnosis (contribution of VE and VGE to VP) might have resulted in the study’s findings not passing the Bonferroni correction. Taking all the above concerns into account, an indirect and comprehensive assessment for VE and VGE could be viewed as the influence of underdiagnosis. This notion emphasizes the importance of considering VE and VGE in investigating the impact of phenotypes and diseases. It can be assumed that the causal relationship exists in the real world if VE and VGE were taken into account because it is not surprising that the circadian rhythm in our daily life would be easily influenced by many factors, such as children busy with morning classes and adults trapped by working hours. In other words, it is good news for us that making a behavior change would be useful for preventing RMFG-related diseases.

But the present study has several limitations: (1) the participants enrolled were all European, hence, whether the relationship between morningness and the RMFG is causal or not in other populations remains unknown; and (2) the study only reported the variates of the cortical structure in the morning person, but the underlying mechanisms warrant further investigation.

Our results provide a clue for researchers to explore the relationship between circadian rhythm and other neuropsychiatric disorders, especially focusing on the specific gyrus of the circadian rhythm. Future studies should focus on the mechanism of how circadian rhythm acts on the RMFG or designing RCTs for circadian treatment modalities to prevent or treat RMFG-related neuropsychiatric disorders.

## Data availability statement

Publicly available datasets were analyzed in this study. This data can be found here: the exposure data for the top 10,000 morningness-related SNPs of the GWAS summary statistics can be accessed at: https://static-content.springer.com/esm/art%3A10.1038%2Fncomms10448/MediaObjects/41467_2016_BFncomms10448_MOESM584_ESM.txt. The outcome GWAS summary statistics for brain cerebral cortex structure can be accessed at: https://enigma.ini.usc.edu/research/download-enigma-gwas-results/ by applying for the data access.

## Ethics statement

This study used publicly available de-identified data from participant studies that were approved by an ethical standards committee concerning human experimentation. No separate ethical approval was required in this study.

## Author contributions

FY and RL: writing—original draft preparation, conceptualization, and methodology. SH, SR, and BH: formal analysis. JL: resources. LP: writing—review and editing. The guarantor (LP) confirms that all listed authors meet the authorship criteria and that no others meeting the criteria have been omitted. All authors contributed to the article and approved the submitted version.

## Funding

This study was supported by the Health Commission of Hunan Province (Grant no. 202204113461 on FY) and the Natural Science Foundation of Hunan Province (Grant no. 2021JJ70046 on BH).

## Conflict of interest

The authors declare that the research was conducted in the absence of any commercial or financial relationships that could be construed as a potential conflict of interest.

## Publisher’s note

All claims expressed in this article are solely those of the authors and do not necessarily represent those of their affiliated organizations, or those of the publisher, the editors and the reviewers. Any product that may be evaluated in this article, or claim that may be made by its manufacturer, is not guaranteed or endorsed by the publisher.

## Footnotes

^1^www.phenoscanner.medschl.cam.ac.uk
